# Prevalence and risk factors of low back pain among operation room staff at a Tertiary Care Center, Makkah, Saudi Arabia: a cross-sectional study

**DOI:** 10.1186/s40557-016-0089-0

**Published:** 2016-01-29

**Authors:** Moath Bin Homaid, Doaa Abdelmoety, Waleed Alshareef, Amer Alghamdi, Fareed Alhozali, Naif Alfahmi, Wael Hafiz, Abdulrahman Alzahrani, Soha Elmorsy

**Affiliations:** Umm Al Qura University, Makkah, Saudi Arabia; Clinical Research Management Department, King Abdullah Medical City, Makkah, Saudi Arabia; Otolaryngology Resident, King Abdullah Medical City, Makkah, Saudi Arabia; Research Center, King Abdullah Medical City, Makkah, Saudi Arabia; Pharmacology, Faculty of Medicine, Cairo University, Cairo, Egypt

**Keywords:** Musculoskeletal pain, Low back pain, Healthcare worker, Operating room staff

## Abstract

**Background:**

Low Back Pain (LBP) is the commonest musculoskeletal disorder and an important occupational hazard among healthcare workers (HCWs) that peaks among Operating Room (OR) staff. This cross-sectional study aimed to assess the prevalence, characteristics, and risk factors of low back pain among operating room (OR) staff in a tertiary healthcare center in Makkah, Saudi Arabia.

**Methods:**

A 39-item self-administered questionnaire was distributed to all available OR staff. Data about personal, sociodemographic, general risk factors OR specific risky activities, and LBP characteristics were obtained. Descriptive, crosstabs, and univariate and multivariate logistic regression tests were employed.

**Results:**

Out of the 143 distributed questionnaires, 84 % were received. LBP prevalence was 74.2 %. No statistically significant associations were detected between LBP and any of the general risk factors (*p* >0.05). However, most of the OR risky activities were significantly associated with the occurrence of LBP (*p* <0.05) e.g. lifting objects above the waist, rotating torso while bearing weight, transferring patients onto bed or chair, pulling a patient up the bed, and repositioning a patient in bed. These significant associations were preserved after adjustment for gender, perceived stress at work, educational level, and receiving education about LBP. Rest and analgesics were reported to be the most common relievers.

**Conclusions:**

LBP is a common health issue among KAMC OR staff. OR risky activities were found to contribute to this problem. We suggest designing educational interventional programs to teach OR staff the best way to prevent this problem.

## Background

Low Back Pain (LBP) is one of the most common complaints requiring medical attention. It is the most common form of musculoskeletal disorders [1, 2]. It is estimated that over half of the general population will seek medical care for back pain at some point in their lives [3]. Globally, the prevalence of LBP among general population ranges between 15 and 45 % [1, 2, 4, 5]. In Saudi Arabia, the prevalence of LBP among general population is estimated to be 18.8 % according to a single study conducted in Al-Qaseem [6]. Usually females complain more than males from LBP [6–15].

Occupational LBP is a common health problem worldwide. Healthcare workers (HCWs) are at a higher risk of developing LBP due to a variety of factors [16]. This problem is associated with major consequences in terms of disability and frequent absence [16]. LBP might lead to activity limitation and sick leaves for more than 50 % of the nurses [17]. Generally, female gender, advanced age, and high Body Mass Index (BMI) are some examples of risk factors commonly associated with LBP [7–12]. Sport and regular physical activity were found to decrease LBP [18]. Working in a surgical department might be associated with a higher risk of developing LBP compared to other departments as shown in a study by Attar [19]. He found OR staff to have the highest prevalence of LBP as compared to other departments [19]. Also, Al Dajah and Al Daghdi reported the highest prevalence of LBP among Operation Room (OR) staff as compared to other HCWs [17]. Working at the OR carries its own risk for developing LBP due to exposure to additional risk factors e.g. prolonged standing and awkward posture during surgeries [13–16].

In Saudi Arabia, the situation of LBP is not different from that in other parts of the world [6, 17, 19, 20]. A few studies addressed the prevalence of LBP among OR staff in Saudi Arabia [17, 19, 20]. However, the assessment was not comprehensive in terms of details of the pain and associated risk factors. This study aims to assess the prevalence and risk factors of LBP among all categories of OR staff in a tertiary center in Makkah, Saudi Arabia.

## Methods

This cross-sectional study was conducted at King Abdullah Medical City (KAMC), Makkah, Saudi Arabia in the period from 1^st^ to 30^th^ June, 2014. It approached all categories of OR staff from different specialties.

KAMC Institutional Review Board (IRB) approved the study and adequate information was distributed on a sheet given to each invited HCW. The research objectives were explained to each participant separately. The questionnaires distributed in this study showed no personal identifiers and so the confidentiality of participants was maintained.

Data collection was carried out through a 39-item, adapted, self-administered questionnaire that was based on the previous work of Karahan et al. [16]. Adaptation included some spelling and grammar changes, adding and rephrasing some questions concerning work experience at KAMC. This adaptation was carried after piloting the questionnaire. Twenty-seven questions were multiple choice ones including some binary (yes/no) questions. The questionnaire was composed of the following sections:

- Questions concerning personal and sociodemographic information: age, gender, height, weight, specialty, etc.
- Questions concerning general LBP risk factors: smoking, psychological stress, standing time, etc.
- Questions concerning OR specific risky activities: lifting, transferring, or pulling patients or objects, etc.

Questions concerning LBP characteristics: presence of LBP, severity, duration, treatment etc. The questionnaires were numbered before distribution to ensure tracking and to calculate the response rate. Participants’ initials were optional to avoid duplication. All the available OR staff were invited including surgeons, anesthesiologists, nurses, anesthesia technicians, OR technicians, and central sterile supply department (CSSD) staff. The questionnaire was delivered to the surgeons’ departments and clinics if they didn’t have duty on the same day in the OR. For the rest of the OR staff the questionnaires were distributed in the OR after obtaining permission from the head of the OR department.

For the purpose of this study LBP was defined as “pain, muscle tension, or stiffness localized below the costal margin and above the inferior gluteal folds, with or without leg pain (sciatica)” [21].

The sample size was estimated using the Epi Info^TM^ version seven software with the following assumptions: a population size of 175 (the number of OR staff at KAMC), an expected frequency of LBP of 80 % as guided by previous studies, a precision of ± 5 at a 95 % confidence limit [15, 18, 22, 23]. This dictated having a sample of 102 participants at a minimum. Therefore, considering that some staff might not be available or might not respond, we decided to approach all available OR staff. The data was coded and entered into STATA version 11.0. For descriptive statistics, percentages were used for categorical variables and the mean (standard deviation [SD]) or the median and the interquartile range were used for numeric data according to the type of distribution. A logistic regression model was constructed with presence of LBP as the dependent variable and all suspected risk factors as independent ones. Univariate analysis was initially performed for each factor separately, then, a multivariate model was built for each risky OR activity while adjusting for demographic and other general risk factors found to give a P value < 0.1 in univariate analysis. A two-sided α was set at 0.05 for all comparative analyses.

## Results

One hundred and forty three (143) questionnaires were distributed and 120 were received with a response rate of 84 %. The mean age of respondents (SD) was 33.9 (7.6) and 89 (74.2 %) of them were males and 31 (25.8 %) were females. Table 1 shows the participants’ characteristics. The prevalence of LBP among all participants collectively was 74.2 %. The prevalence among males was 69.7 % and was 87.1 % among females. Anesthesia technicians and Anesthesiologists had the highest prevalence of LBP followed by nurses, surgeons, CSSD staff, and OR technicians, none of these differences however was statistically significant (Table 1). Out of those who have had LBP, 56.3 % experienced LBP for the first time before joining KAMC while the remaining 43.7 % experienced LBP for the first time after joining KAMC (Table 2).

**Table 1:** Characteristics of study participants

Variable	*n*	%^*^	Have experienced LBP	
			*n*	%^**^
Total	120	100	89	74.2
Age category (*n* = 120)				
< 24	10	8.3	8	80
25 – 34	56	46.7	38	67.9
35 – 44	36	30	27	75
> 45	18	15	16	88.9
Gender (*n* = 120)				
Female	31	25.8	27	87.1
Male	89	74.2	62	69.7
Marital status (*n* = 120)				
Single	30	25	23	76.7
Married	90	75	66	73.3
BMI (*n* = 90)				
Underweight (<18.49)	2	2.2	2	100
Normal (18.5 – 24.99)	27	30	19	67.9
Overweight (25 – 29.99)	37	41.1	24	66.7
Obese (>30)	24	26.7	19	79.2
Educational level (*n* = 119)				
Diploma	21	17.6	12	57.1
Bachelor	41	34.5	31	75.6
Post-Graduate Degree	57	47.9	45	78.9
Specialty (*n* = 119)				
Surgeon	44	37	31	70.5
Anesthesiologist	17	14.3	14	82.4
Anesthesia Technician	12	10.1	10	83.3
Nurse	34	28.6	26	76.5
OR Technician	7	5.9	3	60
CSSD staff	5	4.2	4	57.1
If surgeon, specify: (*n* = 44)				
Breast oncology	2	4.5	1	50
General surgery	9	20.5	5	55.6
Gynecological oncology	2	4.5	2	100
Thoracic	1	2.3	1	100
Urology	3	6.8	3	100
Vascular	2	4.5	1	50
Ophthalmology	6	13.6	3	50
ENT	5	11.4	4	80
Neurosurgery	2	4.5	1	50
Spine	3	6.8	2	66.7
Maxillofacial	1	2.3	1	100
Cardiac	7	15.9	6	85.7
Other	1	2.3	1	100
Current work experience (*n* = 119)				
1 – 5 years	40	33.6	27	67.5
6 – 10 years	33	27.7	24	72.7
> 10 years	46	38.7	37	80.4
Current KAMC OR experience (*n* = 116)				
< 1 year	35	30.2	25	71.4
1 – 2 years	30	25.9	20	66.7
> 2 years	51	44	41	80.4

*LBP* low back pain, *BMI* Body Mass Index, *OR* operating room, *KAMC* King Abdullah Medical City

n = the number of responders to each question

^*^: shows the percentages of respondents in that category out of total respondents

^**^: shows the percentages of those with LBP within each category

**Table 2:** Descriptive data about LBP management (*n* = 89)

Variable	N	% of those with LBP
First LBP (*n* = 87)		
Before joining KAMC	49	56.3
After joining KAMC	38	43.7
Other musculoskeletal pain (*n* = 120)	68	82.4
Severity of LBP		
Mild	32	36
Moderate	48	53.9
Sever	7	7.9
Very sever	2	2.2
Sought medical care	22	24.7
Received a diagnosis (*n* = 22)	18	72.2
used treatment (*n* = 87)	35	39.8
Best LBP reliever: (*n* = 53)		
Rest (*n* = 35)	45	51.72
Medication (*n* = 36)	38	43.68
Physiotherapy (*n* = 35)	8	9.2
Herbs (*n* = 35)	1	1.1
Other (*n* = 35)	3	3.45
LBP has an impact on daily life activities (*n* = 81)	32	39.5
LBP has an impact on work life (*n* = 79)	33	41.8

*n* = the number of respondents to each question if less than 89, N = number of yes answers to each question or item

*LBP* low back pain, *KAMC* King Abdullah Medical City

Table 3 shows the relationship between some common risk factors and LBP. Female gender, advancing age, more years at work, and perceived stress at work were associated with a higher prevalence of LBP. However, none of the associations was statistically significant.

**Table 3:** Association between common risk factors and LBP

Risk factors	*n*	Univariate regression analysis	
		OR (95 % CI)	*P* value
Age	111		
< 24		1	
25 – 34		0.500 (0.059 – 4.232)	0.525
35 – 44		0.264 (0.055 – 1.273)	0.097
> 45		0.375 (0.072 – 1.957)	0.245
Gender	120		
Female		1	
Male		2.940 (0.937 – 9.221)	0.065
Educational level	119		
Diploma		1	
Bachelor		0.356 (0.122 – 1.040)	0.059
Post-graduate degree		0.827 (0.318 – 2.150)	0.696
Specialty	119		
Surgeon		1	
Anesthesiologist		1.788 (0.350 – 9.138)	0.485
Anesthesia technician		3.500 (0.499 – 24.558)	0.208
Nurse		3.750 (0.445 – 31.621)	0.224
CSSD		2.437 (0.448 – 13.260)	0.303
OR technician		1.125 (0.109 – 11.595)	0.921
Working years	119		
1 – 5		1	
6 – 10		0.505 (0.189 – 1.352)	0.174
> 10		0.649 (0.225 – 1.867)	0.422
Working duration at KAMC	116		
< 1 year		1	
1 – 2 year(s)		0.610 (0.223 – 1.670)	0.336
> 2 years		0.488 (0.175 – 1.362)	0.171
Smoking	120		
Yes		1	
No		0.472 (0.175 – 1.275)	0.139
BMI	87		
Underweight /Normal		1	
overweight		1.000 (0.347 – 2.882)	1.000
Obese		1.900 (0.534 – 6.760)	0.322
Received education about LBP	119		
Yes		1	
No		0.455 (0.192 – 1.077)	0.073
Perceived stress level in work environment	116		
Mild		1	
Moderate		1.333 (0.525 – 3.388)	0.545
Sever/ Very sever		4.500 (0.867 – 32.345)	0.073
Standing time	120		
1 – 4 h		1	
5 – 8 h		1.263 (0.343 – 4.647)	0.726
> 8 h		1.417 (0.431 – 4.658)	0.566
Sitting time	118		
1 – 4 h		1	
> 5 h		1.780 (0.483 – 6.568)	0.387
Exercise	120		
Yes		1	
No		0.969 (0.424 – 2.218)	0.941

*n* = the number of respondents to each question

*KAMC* King Abdullah Medical City, *BMI* Body Mass Index

A univariate logistic regression showed that the following OR risky activities were significantly associated with the occurrence of LBP (*P* < 0.05): lifting objects above the waist, rotating torso while bearing weight, transferring patients onto bed or chair, pulling patients up the bed, and repositioning a patient in bed (Table 4). The association between LBP and the following activities, however, did not reach statistical significance (*P* > 0.05): transferring patients onto stretcher, bending to lift item from floor level, and ambulating patients. Adjusting the association of individual OR risky activities for gender, educational level, perceived stress at work, and receiving education did not greatly change the significance of the association. In none significant associations (bending to lift item from floor level and ambulating patients) remained as such. Association between LBP and lifting objects above the waist, rotating torso while bearing became more significant. While the association of LBP with pulling patients up the bed, and pulling patients up the bed became slightly less significant. However, transferring patients onto bed or chair and repositioning a patient in bed, did not show any changes in P value after the adjustment (Table 4).

**Table 4:** The relationship between OR risky activities and LBP

Risk factors	Univariate logistic regression			Multivariate logistic regression (Adjusted)		
	*n*	OR (95 % CI)	*P* value	*n*	OR (95 % CI)	*P* value
Lifting objects above the waist	115	3.06 (0.119 – 0.787)	0.014^*^	109	4.910 (1.500 – 16.00)	0.008^*^
Rotating torso while bearing weight	111	4.490 (1.250 – 16.15)	0.021^*^	105	9.080 (1.950 – 42.28)	0.005^*^
Bending to lift an item from floor level	116	1.590 (0.640 – 3.660)	0.278	110	1.610 (0.600 – 4.290)	0.341
Transferring patients onto bed or chair	117	2.440 (1.010 – 5.910)	0.047^*^	111	2.820 (1.010 – 7.840)	0.047^*^
Transferring patients onto a stretcher	117	2.270 (0.980 – 5.220)	0.055	111	3.310 (1.160 – 9.470)	0.025^*^
Ambulating a patient	115	0.780 (0.300 – 2.040)	0.619	109	0.590 (0.180 – 1.740)	0.315
Pulling a patient up the bed	118	2.630 (1.140 – 6.090)	0.024^*^	112	3.000 (1.090 – 8.250)	0.033^*^
Repositioning a patient in bed	118	2.470 (1.060 – 5.720)	0.035^*^	112	2.820 (1.070 – 7.380)	0.035^*^

Adjusted: Adjusted for gender, perceived stress at work, educational level, and receiving education about LBP

*n* = the number of respondents to each question

^*^ = statistically significant

The description of LBP and its management revealed that the affected participants were complaining mostly from mild to moderate LBP, 36 and 53.9 %, respectively (Table 2). A quarter of the affected participants have sought medical care and 72.7 % of them received a diagnosis (Table 2). Rest and analgesics were reported to be the most effective relievers of LBP (Table 2).

## Discussion

In this study 74.2 % of OR staff at KAMC (including: surgeons, anesthesiologists, nurses, anesthesia technicians, OR technicians, and CSSD) reported to have complained from LBP at some point of their career. This high percentage didn’t exceed the worldwide prevalence, which is reported to be around 84 % [15, 20, 22]. Females in our study were found to complain more from LBP as compared to males. This is also consistent with all the studies in this topic [15, 20, 22, 23].

Globally, among all HCWs, the nurses and physical therapists were found to have the highest prevalence of LBP while secretaries and hospital aids have the lowest [16]. In the present study, the prevalence among anesthesiologists and anesthesia technicians was high, that might be due to the long sitting time and psychological stress in such an advanced tertiary care center. Lifetime prevalence of LBP among nurses ranged between 70 and 80 %, annual prevalence ranged between 15 and 45 %, point prevalence was 30 % [13]. In our study, we found the highest prevalence of LBP among anesthesiologists and anesthesia technicians (82.4 and 83.3 % respectively). Additionally, we found that 76.5 % of the OR nurses and 57.1 % of the OR technicians complain from LBP. This is less than the LBP prevalence among OR nurses and OR technicians that was found to be 84.4 % in a multicenter study conducted in Taif, Saudi Arabia [20]. These values are generally comparable to the worldwide reported values for prevalence of LBP among OR nurses that range between 70.6 and 84 % and the prevalence among OR technicians which hovers around 84 % [15, 20, 22]. We have also found that 70 % of the surgeons at KAMC complain from LBP. It is a high percentage but still less than that reported in studies conducted in some other countries. For instance 84.8 % of surgeons were found to complain from LBP according to a study conducted in Iran [15]. Statistically, however, the relationships between LBP and specialty, psychological stress, and sitting and standing time did not reach significance. Further studies with larger samples of HCWs may be needed to confirm the observed associations and to illustrate the possible causes.

Prolonged standing and sitting, awkward posture during surgeries, work overload, psychological stress, physically hard work, and long working hours may predispose to LBP. Smoking, high BMI, advancing age, female gender, inactivity, long standing time, and perceived stress were significantly associated with the presence of LBP worldwide and in Saudi Arabia [7–12]. However, we did not find statistically significant relationship between LBP and gender, age, BMI, regular exercise, standing time, specialty, or work experience in this study.

OR staff usually perform certain risky activities on daily basis that were found to significantly associate with LBP [16]. This may include lifting heavy objects above the waist, transferring patients onto bed or chair, transferring patients onto a stretcher, ambulating a patient, repositioning patients, pulling a patient up the bed, and rotating torso while bearing some weight [16]. In the present study, we have found that some of such activities were indeed significantly associated with the presence of LBP as consistent with the other studies. These association should shed light on the importance of enrolling OR staff in some educational interventional programs on how to lift objects and how to deal with various satiations that might face the them. Weight reduction programs can also be addressed to improve the OR staff quality of life.

The participants in this study have reported that rest and Analgesics are the best LBP relievers. Studies on the same topic have drawn the same conclusion.

LBP is common among OR staff at KAMC. Risky activities were found to contribute significantly to the problem. Rest and analgesics were reported to be the most common relievers of LBP.

A larger sample size including OR staff from different centers is needed to achieve more precise and comprehensive results. The sampling was based on including all the available OR staff at KAMC and the study was conducted at summer time in which many of the OR staff were on vacation.

## Conclusion

Educational programs are needed for the OR staff to teach them the best way to prevent this problem. Such programs may include practical sessions on how to lift and pull heavy objects as well as some exercises that could be carried out during work. We also recommend to enroll the OR staff in stress management courses. It is also important to consider the shoes that a staff wears during work. Enhancing sports activities and designing programs to encourage weight reduction may also help. Future prospective randomized studies will be needed to evaluate such educational programs in order to find the best way to solve the problem and to improve the OR staff quality of life.

## Abbreviations

BMI: Body Mass Index
CSSD: Central Sterile Supply Department
HCW: Healthcare worker
IRB: Institutional Review Board
KAMC: King Abdullah Medical City
LBP: Low Back Pain
OR: Operating Room
SD: Standard Deviation
